# Psychometric validation of the Arabic self-directed ageism scale among Lebanese middle-aged and older adults

**DOI:** 10.3389/fpsyg.2026.1911928

**Published:** 2026-09-16

**Authors:** Charbel Ziade, Maria-Theresa Chams, Rudy S. Younes, Chadia Haddad, Elias Rahme

**Affiliations:** 1 Department of Psychology and Social Sciences, Faculty of Arts and Sciences, Holy Spirit University of Kaslik (USEK), Jounieh, Lebanon; 2 IDEES Multidisciplinary Research Group, Faculty of Arts and Sciences, Holy Spirit University of Kaslik (USEK), Jounieh, Lebanon; 3 INSPECT-LB (Institut National de Santé Publique, d’Épidémiologie Clinique et de Toxicologie-Liban), Beirut, Lebanon; 4 Research Department, Psychiatric Hospital of the Cross, Jall Eddib, Lebanon

**Keywords:** Arabic adaptation, Lebanon, older adults, psychometric validation, self-directed ageism, internalized ageism

## Abstract

**Background:**

Self-directed ageism is the internalization of negative societal stereotypes about one’s own aging. This concept has been linked to poorer mental health, diminished self-esteem, and reduced quality of life among the older population. Despite its relevance, there is currently no validated Arabic tool to measure this construct in Arab populations. This study aimed to validate the first Arabic version of the self-directed ageism scale in a Lebanese sample of middle-aged and older adults.

**Methods:**

The original scale was translated into Arabic using the forward-backward translation method. It was then administered to a total of 223 community-dwelling and institutionalized middle-aged and older Lebanese adults alongside the Arabic version of the Rosenberg Self-esteem scale, the negative subscale of the Arabic version of the Kogan Attitudes Toward Older People scale, and the QOLS-6 scale, which was used to measure quality of life.

**Results:**

Exploratory and confirmatory analyses supported a unidimensional structure of the 11-item Arabic self-directed ageism scale. Furthermore, the scale presented high internal consistency (Cronbach’s α = 0.889) and convergent validity was partially supported through significant associations with self-esteem (*r* = −0.547, *p* < 0.001), negative attitudes towards older people (*r* = 0.205, *p* = 0.002) and quality of life (*r* = 0.288, *p* < 0.001).

**Conclusion:**

The Arabic self-directed ageism scale is a reliable instrument to measure self-directed ageism. This study provides the first quantitative tool to capture this phenomenon among Arabic-speaking populations.

## Introduction

Population aging is a defining demographic trend of the 21st century (Sabri et al., 2022). This has been accompanied by a growing recognition of the psychological and social challenges faced by older adults. The aging experience worldwide is shaped by broader challenges to quality of life, social participation, and emotional wellbeing (Leung et al., 2021; Phillips and Gyasi, 2021). Healthy aging frameworks emphasize physical health and continued engagement, including strong social networks, participation in physical activity, and independence in daily living, as key protective factors (Abud et al., 2022).

In Arab countries, and in Lebanon specifically, these challenges are further shaped by region-specific social and cultural dynamics (Sibai et al., 2018). Recent evidence suggests that loneliness and social isolation are growing concerns among older adults in the region, consistently associated with poorer quality of life and worsened mental health (Younes et al., 2026). Positive self-perceptions of aging and greater intergenerational contact have been linked to better attitudes towards older adults (Cigarroa et al., 2025), while sociodemographic and educational factors also shape these attitudes, underscoring the widespread nature of ageism (Yáñez-Yáñez et al., 2022).

Among these, ageism has emerged as a critical concern (Marques et al., 2020; Berrío Valencia, 2012). Ageism is defined as assumptions, biases, and discriminatory actions toward an individual or a group based on their age (Officer and de la Fuente-Núñez, 2018). This shift has become a tangible social issue that affects health, well-being, and human rights across the lifespan (World Health Organization, 2021). It is important to note that ageism can become internalized, as individuals may start to believe in society’s negative views on aging and integrate them into their own belief systems (Ishikawa, 2023). This phenomenon is known as self-directed ageism and refers to the assimilation of negative social stereotypes about aging and the application of these ideas to oneself, affecting self-perception and well-being (Henry et al., 2023). This internalization process does not only begin in old age, but could also accumulate throughout the lifespan, with middle-aged adults absorbing and internalizing ageist beliefs before they become relevant in later life (Kornadt et al., 2017; Levy, 2009). Indeed, during middle adulthood, individuals begin to approach late adulthood, experiencing shifts in self-perception and life priorities. They tend to have heightened self-evaluation of societal expectations of productivity and youth (Erikson, 1963, 1982; Chen et al., 2021). This period also has several markers of aging, as middle-aged adults begin anticipating retirement, notice early declines in physical health, and often witness their children growing up, all of which can heighten awareness of their own aging (Dutt et al., 2018; Lee et al., 2004; Levinson, 1986; Solhi et al., 2022; Westerståhl et al., 2025). This aligns with theoretical accounts suggesting that latent age stereotypes increasingly serve as a template for the aging self, starting in midlife and slowly gaining more relevance in shaping how individuals perceive the aging process before old age is reached (Brothers et al., 2021; Kotter-Grühn and Hess, 2012). Therefore, middle age represents an important period for the early consolidation process of self-directed ageism (Davis and Friedrich, 2010). In this sense, ageism has two forms: other-directed ageism and self-directed ageism.

Self-directed ageism has been documented worldwide and has been associated with a range of negative health and psychological outcomes (Tully-Wilson et al., 2021; Vanleerberghe et al., 2024). Among these outcomes, self-esteem is a relevant construct as self-directed ageism has been linked to lower self-worth and a reduced sense of personal value (Marquet et al., 2019; Özcan Tozoğlu and Gürbüzer, 2025). Beyond self-esteem, self-directed ageism is also associated with poorer mental health, and overall quality of life (Vanleerberghe et al., 2024; Christian and Halliday, 2025). Moreover, self-directed ageism seems particularly noteworthy within Arab populations. Indeed, recent systematic reviews of the literature have shown that ageism is prevalent in Arab countries, especially in low and middle-income contexts such as Lebanon (Younes and Rajha, 2025; Younes and Abboud Mzawak, 2026). One review further suggested that higher levels of ageism on the social level may contribute to its internalization, leading to self-directed ageism among older people (Younes and Abboud Mzawak, 2026).

Self-directed ageism remains understudied in Arab communities, especially through quantitative research. Current research on self-directed ageism in Arab countries has mostly relied on qualitative approaches (Abi Chahine and Kienzler, 2022). While these provide valuable insights, they do not allow for generalization or for examining relationships between self-directed ageism and other psychological outcomes. Considering the prominence of ageism in Arab culture, the absence of a validated quantitative tool represents a significant gap and limits researchers’ ability to assess its prevalence, severity, and consequences (Boateng et al., 2018).

In terms of quantitative research, despite the existence of a validated and measurable English-language scale (Vanleerberghe et al., 2024), no equivalent tool has been culturally and linguistically adapted for Arabic-speaking populations (Younes and Abboud Mzawak, 2026). For instance, Alquwez et al. (2018) validated an Arabic version of the Kogan Attitudes toward Older People Scale among a Saudi sample, which captures general attitudes toward older adults but does not assess internalized or self-directed ageism. A more recent exception is Li X. et al. (2026), who validated an Arabic version of the WHO Ageism Experiences Scale in a Libyan humanitarian context, which includes a self-directed ageism subscale. However, confirmatory factor analysis of this subscale did not meet conventional criteria for good model fit, and the subscale is limited to only five items, which limits the scale from comprehensively capturing the construct. On the other hand, the scale used in the present study assesses self-directed ageism through 11 items, allowing for a more thorough measurement of the construct. The self-directed ageism scale, developed and validated in Belgium, has demonstrated strong psychometric properties, including good reliability and construct validity (Vanleerberghe et al., 2024). However, its applicability to Arab populations remains uncertain without proper investigation.

Considering the importance of advancing research on self-directed ageism, particularly in Arab countries, the objective of this study is to validate, translate and culturally adapt the Arabic version of the self-directed ageism scale in a Lebanese sample of middle-aged and older adults since they represent the population that is mostly affected by internalized ageist beliefs (Levy, 2009; Ishikawa, 2023). This aims to provide a reliable and valid tool for quantitative research, enabling researchers to measure self-directed ageism in quantitative studies and population surveys. Ultimately, the availability of a validated scale would fill an important gap in the literature on ageism. We hypothesize that the Arabic version of the self-directed ageism scale will demonstrate a factorial structure consistent with the original scale, along with adequate reliability and convergent validity with related constructs among Lebanese middle-aged and older adults.

## Methodology

### Participants and procedure

Two modalities were used to recruit participants, institutionalized older adults were administered the questionnaire directly in nursing homes, and community-dwelling participants were recruited using convenience sampling through an online Google Forms survey given the practical challenges of accessing a random, nationally representative sample of older adults in Lebanon. The inclusion criteria were being Arabic speaking, Lebanese, residing in Lebanon, and aged 40 or older.

### Ethical approval

This study earned ethical approval through the Higher Center for Research at the Holy Spirit University of Kaslik (USEK; HRPP/202610/FT/0115). Data were collected between February 6th and March 1st, 2026. Informed consent was obtained from all study participants prior to data collection. For nursing home residents, the research team facilitated the informed consent process by explaining the study’s objectives to potential participants and ensuring their voluntary participation. Participation was voluntary, and participants had the right to withdraw at any time without their data being retained. All data were collected anonymously and treated with strict confidentiality.

### Measures

#### self-directed ageism scale

The self-directed ageism scale is a psychometric tool designed to assess self-directed ageism among older adults. For this study, the self-directed ageism scale, validated by Vanleerberghe et al. (2024), was translated into Arabic using the forward-backward method (Beaton et al., 2000) to ensure accuracy and cross-cultural adaptation. This scale consists of 11 statements assessing internalized ageist beliefs. The full survey which includes the Arabic version of this scale, was pilot-tested on 15 Arabic-speaking Lebanese individuals to ensure linguistic clarity and cultural appropriateness. Participants were asked to review each item for comprehension and to provide any comments or feedback, and no comprehension issues or comments were reported. As no comprehension issues or comments were reported, data collection proceeded as planned without further modifications to the questionnaire. The present study used the 11-item self-directed ageism scale developed by Vanleerberghe et al. (2024) rated on a 5-point Likert scale (1- strongly disagree, 5- strongly agree) which has a range of 11–55, with higher scores demonstrating higher levels of self-directed ageism. Item 8 (‘Compared to other older people I’m very lucky’) was reverse scored, so that higher scores consistently reflect higher levels of self-directed ageism. The scale previously demonstrated good internal consistency with a Cronbach’s alpha of 0.898.

#### Quality of life scale (QOLS-6)

The QOLS-6 was originally developed by Phillips et al. (2002) to assess the quality of life in six dimensions over the preceding month: physical, psychological, economic, occupational, familial relationships, and social interactions. This scale was translated into Arabic using the forward and backward technique to ensure accuracy and cross-cultural adaptation. Items are rated on a 5-point Likert scale, ranging from 1 (very poor) to 5 (excellent), with the total score in the range of 6–30, where higher scores reflect a better quality of life. Huang et al. (2025) showed that QOLS-6 had good reliability and validity in China, demonstrating a Cronbach’s alpha of 0.865.

#### Rosenberg self-esteem scale (RSES)

The Rosenberg Self-Esteem Scale (RSES) is a psychometric instrument designed to assess an individual’s overall self-worth by measuring both positive and negative feelings about the self (Rosenberg, 1965). The Rosenberg Self-Esteem Scale (RSES) is the most widely used instrument to measure self-esteem in social science research (Sinclair et al., 2010). Items are rated on a 4-point Likert scale, ranging from 0 to 3(0-strongly disagree, 3-strongly agree), with a total score ranging between 0 and 30, with higher scores indicating higher levels of self-esteem. For this scale, a previously validated Arabic version was used (Ben Ayad et al., 2024), with the RSES demonstrating acceptable reliability and validity, with a Cronbach’s alpha of 0.70.

#### Kogan attitudes toward older people scale: negative subscale

The KAOP scale was developed by Kogan (1961) in an American population, to assess the attitudes towards aging. This scale consists of 34 items; half are negatively worded, and half are positively worded. Similar to a previous study in Iraq and to not overwhelm participants with an exhaustive questionnaire, the present study only used negatively worded items to assess negative attitudes toward older people (Ghanem, 2019), as negative attitudes were the most relevant for this study. The scale uses a 6-point Likert scale (1- strongly disagree; 6- strongly agree), in the context of this study, higher scores demonstrate higher negative attitudes towards older people. In the present study, a validated Arabic version (Alquwez et al., 2018) was used, and the KAOP scale demonstrated good reliability and validity, with a Cronbach’s alpha of 0.89.

### Translation and adaptation procedures

In the present study, the Quality-of-Life Scale (QOLS-6) and the self-directed ageism scale were translated following the standard forward–backward translation method to ensure linguistic and conceptual equivalence (Beaton et al., 2000). First, the original English versions of the scales were translated into Arabic by a bilingual expert (forward translation). Then, a second independent bilingual translator, blinded to the original versions, translated the Arabic versions back into English (backward translation). The two English versions were compared to identify and resolve any discrepancies, ensuring accuracy and consistency of meaning. In addition, the translated versions were reviewed by specialized psychologists to ensure clarity, cultural relevance, and to eliminate any potential ambiguity in the items.

### Statistical analysis

Data were analyzed using SPSS version 27.0. Categorical variables were summarized as frequencies and percentages, while continuous variables were presented as means and standard deviations. The normality of the self-directed ageism scale was assessed through visual inspection of histograms, as well as skewness and kurtosis values, with acceptable thresholds defined as values below ±2. The distribution showed slight right skewness; however, skewness and kurtosis values remained within acceptable limits, indicating approximate normality.

Exploratory factor analysis (EFA) was conducted on the entire sample using principal component analysis with varimax rotation to assess the dimensionality of the 11-item self-directed ageism scale and to examine whether the factor structure previously reported in the literature was reproduced in the present Lebanese Arabic sample. PCA was used as an exploratory assessment of the scale structure. The suitability of the data for factor analysis was evaluated using the Kaiser–Meyer–Olkin (KMO) measure and Bartlett’s test of sphericity. Confirmatory factor analysis (CFA) was conducted using AMOS to evaluate the factor structure identified in the exploratory factor analysis (EFA) on a random half of the sample (*n* = 116). Model fit was assessed using the chi-square statistic divided by degrees of freedom (*χ*^2^/df), with values <2 indicating good fit and values <5 indicating acceptable fit. The Root Mean Square Error of Approximation (RMSEA) was interpreted as indicating a close fit when <0.05 and an acceptable fit when <0.08–0.10. The Comparative Fit Index (CFI), Tucker–Lewis Index (TLI), Goodness-of-Fit Index (GFI), and Adjusted Goodness-of-Fit Index (AGFI) were also used, with values ≥0.90 considered indicative of acceptable model fit. Also, the composite reliability (CR) and average variance extracted (AVE) were calculated to assess construct reliability and convergent validity, respectively. Internal consistency was evaluated using Cronbach’s alpha.

Given the slight skewness of the self-directed ageism scale, Spearman’s correlation coefficients were used to examine unadjusted bivariate associations between self-directed ageism and related variables. To further assess the association between self-directed ageism and quality of life after accounting for potential confounding variables, a partial correlation analysis was performed, adjusting for age, gender, education, financial situation, health problems, self-esteem, attitudes toward older people, employment status, and current housing situation. Group comparisons were performed using an independent-samples t-test for comparisons between two groups and one-way analysis of variance (ANOVA) for comparisons across more than two groups. Cohen’s d was used for comparisons between two groups, while omega squared (ω^2^) was used to estimate effect size for ANOVA. Pearson’s r was reported for correlation analyses, and 95% confidence intervals were reported for effect estimates where applicable.

Finally, Multiple linear regression analysis was performed to identify independent factors associated with self-directed ageism. The assumptions of the model were examined through inspection of the regression plots. The normal probability plot (P–P plot) indicated that the residuals were approximately normally distributed, as they closely followed the diagonal line. A *p*-value < 0.05 was considered statistically significant.

## Results

### Sample description

The study included 223 participants, with a slight predominance of males (53.8%). The mean age was 70.43 ± 11.25 years (range: 40–93 years), with nearly half of the participants (49.3%) aged between 70 and 79 years. More than half of the sample had a primary level of education (53.8%), and most participants were retired (78.5%), and 62.8% of them reported dissatisfaction with their financial situation. Also, the majority resided in nursing homes (76.7%) and most of them reported having health problems (82.5%) (Table 1).

**Table 1:** Sociodemographic and health characteristics of the study population (*N* = 223).

Variable	Category	*n* (%)
Gender	Male	120 (53.8%)
	Female	103 (46.2%)
Education level	Illiterate	3 (1.3%)
	Primary	120 (53.8%)
	Secondary	38 (17.0%)
	Technical	10 (4.5%)
	University	52 (23.3%)
Employment status	Retired	175 (78.5%)
	Unable to work	2 (0.9%)
	Unemployed	11 (4.9%)
	Employed full-time	25 (11.2%)
	Employed part-time	10 (4.5%)
Financial situation satisfaction	Not at all satisfied	15 (6.7%)
	Not satisfied	140 (62.8%)
	Neutral	28 (12.6%)
	Satisfied	37 (16.6%)
	Very satisfied	3 (1.3%)
Current housing situation	Living alone	2 (0.9%)
	Living with family	50 (22.4%)
	Nursing home	171 (76.7%)
Health problems	No	39 (17.5%)
	Yes	184 (82.5%)
Age category (years)	40–59	40 (17.9%)
	60–69	29 (13.0%)
	70–79	110 (49.3%)
	≥80	44 (19.7%)
	Mean ± SD	70.43 ± 11.25
	Min–Max	40–93

### Construct validity

The exploratory factor analysis (EFA) using the varimax rotation was presented in Table 2. The results showed a unidimensional structure of the self-directed ageism scale. Sampling adequacy was excellent, as indicated by a Kaiser–Meyer–Olkin (KMO) value of 0.948, and Bartlett’s test of sphericity was statistically significant (*p* < 0.001). The varimax-rotated solution extracted a single factor explaining 62.74% of the total variance. Factor loadings ranged from 0.547 to 0.916, indicating satisfactory to excellent item contributions to the underlying construct. Communality values ranged from 0.300 to 0.838, suggesting that most items were well represented by the extracted factor.

**Table 2:** Exploratory factor analysis results of the self-directed ageism: Varimax-rotated solution.

Factor	Item	Factor 1	H2 communalities
I have this feeling that the aged no longer count these days	5	0.916	0.838
I have the feeling that the aged often are being considered less important or treated unfairly compared to other groups of people	9	0.896	0.803
Older people should have much more of a say in what is being organized for them	6	0.874	0.763
Some people act like I do not have anything left to contribute to society now that I’m older	4	0.866	0.750
Society is especially focused on youngsters; the interests of older people are not taken into account	3	0.848	0.720
When times get rough, older people usually suffer worst	1	0.795	0.632
Since I’m older, I have regularly noticed that people no longer take me seriously	7	0.793	0.628
I find it hard to swallow to be an older person	10	0.776	0.603
Older people are a separate group in society with their own interests	2	0.699	0.489
Compared to other older people I’m very lucky	8	0.613	0.376
Today’s older people have more difficulties than in the past	11	0.547	0.300
Cronbach alpha value = 0.889			
Total variance explained 62.74%			
Kaiser-Meyer-Olkin (KMO) = 0.948			
Bartlett’s test of sphericity *p* < 0.001			

The scale demonstrated high internal consistency, with a Cronbach’s alpha coefficient of 0.889, supporting the reliability of the self-directed ageism measure.

The confirmatory factor analysis (CFA) conducted on the randomly selected 50% sample supported the adequacy of the one-factor structure of the self-directed ageism scale. The model demonstrated an acceptable overall fit (χ^2^/df = 1.916, RMSEA = 0.089 [95% CI: 0.060–0.118], CFI = 0.962, TLI = 0.952, GFI = 0.882, AGFI = 0.823), providing overall support for the unidimensional structure, although some fit indices suggest a moderate rather than excellent fit in the validation subsample. Also, the scale demonstrated high composite reliability (CR = 0.949), indicating strong internal consistency. The average variance extracted (AVE) was 0.634, exceeding the recommended threshold of 0.50 and supporting adequate convergent validity (Table 3).

**Table 3:** Confirmatory factor analysis fit indices for the self-directed ageism scale in the randomly selected 50% sample.

CFA model of the self-directed ageism scale	χ^2^/df	The Steiger-Lind RMSEA [95% CI]	CFI	TLI	GFI	AGFI	Factor loading range	CR	AVE
Self-directed ageism scale	1.916	0.089 [0.060; 0.118]	0.962	0.952	0.882	0.823	0.521–0.919	0.949	0.634

χ^2^/df, chi-square to degrees of freedom ratio; RMSEA, root mean square error of approximation; CI, confidence interval; CFI, comparative fit index; TLI, Tucker–Lewis index; GFI, goodness-of-fit index; AGFI, adjusted goodness-of-fit index; CR, composite reliability; AVE, average variance extracted.

Figure 1 presents the CFA results of the self-directed ageism scale. The model supports a unidimensional structure, with all items loading significantly on the latent construct (*p* < 0.001). The standardized factor loadings ranged from 0.521 to 0.919. Overall, the majority of items demonstrated strong loadings above 0.70, indicating that they are good indicators of the underlying latent construct.

**Figure 1:**
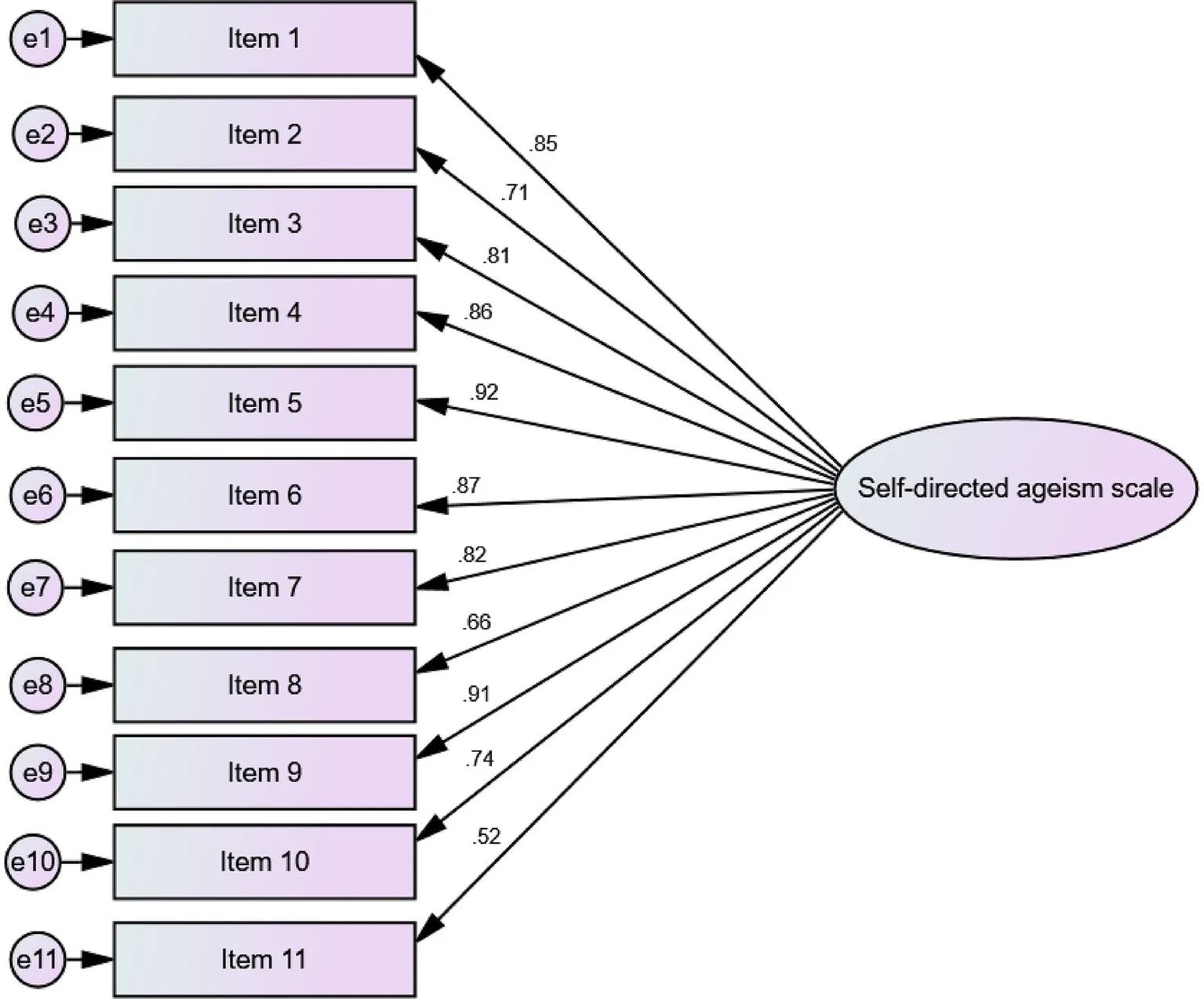
Confirmatory factor analysis path diagram of the self-directed ageism scale conducted on a random 50% sample.

Table 4 presents the item-total statistics for the self-directed ageism scale. The corrected item–total correlations ranged from 0.483 to 0.882, indicating good to excellent homogeneity among items. Most items showed strong correlations (>0.60) with the total score, except item 11, which showed the lowest correlation (0.483). Regarding reliability, Cronbach’s alpha if item deleted ranged from 0.921 to 0.940, which is very close to the expected overall alpha of the scale. Regarding the correlation matrix between the self-directed ageism total scale and its individual items, all items were significantly and positively correlated with the total scale (*p* < 0.01), with coefficients ranging from moderate to very strong (*r* = 0.580 to 0.909) (Supplementary Table S1).

**Table 4:** Corrected item-total correlations and Cronbach’s alpha if item deleted for the self-directed ageism scale.

Self-directed ageism scale items	Corrected item-total correlation	Cronbach’s Alpha if Item deleted
Item 1	0.746	0.928
Item 2	0.633	0.932
Item 3	0.806	0.925
Item 4	0.823	0.924
Item 5	0.882	0.921
Item 6	0.838	0.926
Item 7	0.738	0.928
Item 8	0.542	0.935
Item 9	0.863	0.923
Item 10	0.721	0.929
Item 11	0.483	0.940

### Convergent validity

Table 5 shows the correlations between self-directed ageism, quality of life, self-esteem, and the Kogan scale. Self-directed ageism was significantly and positively correlated with quality of life (*r* = 0.288, *p* < 0.001) and with the Kogan scale (*r* = 0.205, *p* = 0.002), indicating a positive unadjusted association where higher ageism scores were associated with higher scores on these measures. However, after adjustment for the other variables, the association between quality of life and self-directed ageism was negative (B = −0.413, β = −0.090, *p* = 0.022) (Supplementary Table S1). In contrast, a significant negative correlation was observed between self-directed ageism and self-esteem (Rosenberg scale) (*r* = −0.547, *p* < 0.001), suggesting that higher levels of self-directed ageism are associated with lower self-esteem. Internal consistency varied across the convergent measures: the Rosenberg Self-Esteem Scale (RSES) demonstrated good reliability (α = 0.830), while the Quality of Life Scale (QOLS-6; α = 0.642) and the Kogan Attitudes Toward Older People negative subscale (KAOP; α = 0.566) showed lower internal consistency.

**Table 5:** Correlation between self-directed ageism, quality of life (QOL), and Rosenberg Self-Esteem Scale.

Variable	Self-directed ageism	*p*-value
QOL total	0.288	<0.001
Rosenberg total	−0.547	<0.001
Kogan scale	0.205	0.002

Values represent unadjusted Spearman correlation coefficients.

### Bivariate analysis of self-directed ageism across participant characteristics

Table 6 presents self-directed ageism scores according to sociodemographic and health characteristics. No significant difference was observed according to gender (*p* = 0.119). In contrast, significant differences were found across age groups, with higher self-directed ageism scores observed among participants aged 70–79 and ≥80 years compared to younger age groups (*p* < 0.001). Significant variations were also observed according to education level (*p* < 0.001), employment status (*p* < 0.001), financial situation satisfaction (*p* < 0.001), living situation (*p* < 0.001), and presence of health problems (*p* < 0.001). Overall, higher self-directed ageism scores were observed among older participants, those with health problems, those living in nursing homes, and those reporting lower socioeconomic conditions.

**Table 6:** Self-directed ageism scores according to sociodemographic and health characteristics.

Variable	Category	Self-directed ageism scale	Effect size (ω^2^/Cohen’s d)	95% CI	*p*-value
		Mean ± SD			
Gender	Male	47.89 ± 8.91	0.254	−0.011; 0.518	0.119
	Female	45.55 ± 9.54			
Age group (years)	40–59	33.05 ± 9.01	0.553	0.468; 0.619	<0.001
	60–69	43.41 ± 10.82			
	70–79	51.18 ± 3.12			
	≥80	50.64 ± 4.51			
Education level	Illiterate	27.67 ± 14.57	0.300	0.197; 0.385	<0.001
	Primary	50.86 ± 4.36			
	Secondary	41.97 ± 11.15			
	Technical	33.40 ± 8.34			
	University	44.69 ± 10.03			
Employment status	Retired	50.63 ± 4.47	0.618	0.541; 0.676	<0.001
	Unable to work	34.50 ± 12.02			
	Unemployed	34.18 ± 6.87			
	Employed full-time	32.08 ± 10.00			
	Employed part-time	33.10 ± 8.25			
Financial situation	Not at all satisfied	31.73 ± 12.23	0.274	0.172; 0.359	<0.001
	Not satisfied	49.32 ± 6.16			
	Neutral	45.79 ± 9.59			
	Satisfied	45.78 ± 10.00			
	Very satisfied	27.33 ± 6.03			
Living situation	Living alone	38.00 ± 4.24	0.717	0.659; 0.762	<0.001
	Living with family	32.40 ± 8.72			
	Nursing home	51.13 ± 3.03			
Health problem	No	32.31 ± 8.15	−2.741	−3.169; −2.309	<0.001
	Yes	49.88 ± 5.99			

Cohen’s d was used to estimate effect size for comparisons between two groups, whereas omega squared (ω^2^) was used for ANOVA.

### Multivariate analysis of factors associated with self-directed ageism

Table 7 presents the results of the multiple linear regression analysis with self-directed ageism as the dependent variable. The results showed that Living in a nursing home was significantly associated with higher self-directed ageism (Beta = 18.892, *p* < 0.001). Negative attitudes toward older people (Beta = 0.326, *p* < 0.001) and being a female gender (β = 0.068, *p* = 0.046) were also significantly associated with higher self-directed ageism. In contrast, higher quality of life was associated with lower self-directed ageism (Beta = −0.413, *p* = 0.022), as well as better financial situation (Beta = −0.700, *p* = 0.041). Notably, nursing home residents (76.7% of the sample) had a mean self-directed ageism score of 51.13 (SD = 3.03), compared to 32.62 among those not residing in nursing homes. Multicollinearity was assessed using Variance Inflation Factors (VIFs); all VIF values were below 2, indicating no problematic multicollinearity (Table 7).

**Table 7:** Linear regression analysis considering self-directed ageism as the dependent variable.

Predictor	Unstandardized Beta	Standardized Beta	*p*-value	95% confidence level	VIF
Current housing situation (Nursing home)	18.892	0.865	<0.001	17.190, 20.595	1.501
Kogan negative attitude	0.326	0.208	<0.001	0.222, 0.431	1.089
Quality of life (total)	−0.413	−0.09	0.022	−0.766, −0.060	1.449
Financial situation	−0.700	−0.067	0.041	−1.370, −0.031	1.029
Gender (Female vs. male*)	1.254	0.068	0.046	0.024, 2.483	1.088

Variables Entered in the Model: age, gender, education, financial situation, health problems, quality of life, self-esteem, attitudes toward older people, current housing situation and employment status.

Adjusted *R*^2^ = 0.769.

*indicates the reference category for categorical variables.

## Discussion

The present study’s aim was to culturally validate and translate the self-directed ageism scale initially constructed by Vanleerberghe et al. (2024) on a sample of middle-aged and older Lebanese adults. Culturally, the validation of the present scale is significant since Arab cultures have shown high levels of ageism according to recent studies (Younes and Abboud Mzawak, 2026). The negative perception of old age often leads to negative self-views among older adults themselves, who internalize ageist beliefs into self-directed ageism, making the construct important to examine in such contexts, which underlines the importance of developing a scale to measure it (Abi Chahine and Kienzler, 2022; Sanders et al., 2024).

Importantly, Vanleerberghe et al. (2024) and Nieves-Lugo et al. (2021) found self-directed ageism to be associated with worse quality of life outcomes. While these findings stem from Western countries having a sociocultural background and context that is fundamentally different from Arab countries, they serve as important results suggesting the importance of studying self-directed ageism in other contexts. Notably, in Arab countries, no validated quantitative tool exists to capture this phenomenon.

Overall, the self-directed ageism scale presented sound psychometric properties, presenting good internal consistency, a clear unidimensional factor structure, and evidence of convergence validity. Exploratory factor analysis yielded a unidimensional structure for the Arabic self-directed ageism scale, and a single factor accounted for 62.74% of the total variance. Across the 11 retained items factor loading ranged from 0.547 to 0.916, which suggests satisfactory to excellent item-level contributions to the construct. The Kaiser–Meyer–Olkin (KMO) value was 0.948 and a statistically significant Bartlett’s test of sphericity confirm the suitability of the data for factor analysis which exceed the commonly recommended thresholds (Kaiser, 1974), which presents how the items are strongly intercorrelated.

The structure in the present study is unidimensional. It is consistent with the conceptualization of self-directed ageism as a single construct which is related to internalized ageist beliefs linked to negative stereotypes about aging (Levy, 2009). This unidimensional finding is consistent with the final structure presented by the original scale developers in which Vanleerberghe et al. (2024) reached this unidimensional structure after reducing several items. This further suggests that self-directed ageism works as one single internalization process and not as a multidimensional construct, unlike other measures of ageism such as the Fraboni Scale of Ageism (Fraboni et al., 1990) the concept of self-directed ageism is grounded in a single internalization process.

These present results support past theories. For the confirmatory factor analysis a randomly selected 50% subsample also confirmed the one-factor solution, model fit indices were partially acceptable (see Table 3) since both CFI And TLI values exceed 0.95 demonstrating evidence that this model is fit, on the other end the RMSEA value exceeded 0.080 for close fit and approached 0.090, additionally GFI and AGFI were slightly below the 0.90 threshold, it was still within an acceptable range for a moderately sized subsample according to Hu and Bentler (1999). The CFA provided overall support for the one-factor structure, although the RMSEA, GFI, and AGFI values suggested a moderate rather than excellent model fit. These moderate fit indices suggest that while the one-factor structure is broadly supported, the model may not capture the full complexity of the self-directed ageism construct as precisely as indices in the excellent-fit range would imply (Stanley and Edwards, 2016). This underscores the importance of replicating the factor structure in larger and more representative samples before firmly establishing the structural validity of the Arabic version of this scale. High internal consistency was demonstrated for the present scale with a Cronbach’s alpha coefficient of 0.889, exceeding the accepted score usually regarded for strong reliability which is 0.80, also this score seems to be almost identical to the score of the final model obtained in the original scale validation by Vanleerberghe et al. (2024) where a Cronbach’s alpha score of 0.898 was reported, suggesting that the items of the present Arabic version of this scale are consistent with measuring the same construct, furthermore all items demonstrated significant positive correlations with the total scale score (*r* = 0.580 to 0.909), further supporting internal consistency.

Convergent validity was tested by examining the relationships between self-directed ageism and three theoretically relevant constructs that were chosen, namely self-esteem, quality of life, and attitudes toward older people. Concerning self-esteem, as expected based on theoretical expectations (Özcan Tozoğlu and Gürbüzer, 2025; Sanders et al., 2024), higher levels of self-directed ageism were significantly linked to lower levels of self-esteem. This aligns with Levy’s Stereotype Embodiment theory (2009), which postulates that negative internalized ageist beliefs often worsen the individual’s self-worth. It is also in line with Marquet et al. (2019) and Masse and Meire’s (2012) findings, where negative perceptions of aging were linked to lower levels of self-esteem.

Moreover, it was expected that self-directed ageism would be negatively associated with quality of life, as suggested by previous research (Velaithan et al., 2024; Vanleerberghe et al., 2024). However, an unexpected positive association was found, a possible explanation could be related to specific items of the scale that was used for Quality of life measurement which is QOLS-6 (Phillips et al., 2002), particularly items assessing physical health and economic status (item 1: ‘How was your physical health in the last month?’; item 3: ‘How was your economic status in the last month?’). Since a significant part of the sample resided in nursing homes where basic needs are usually met, their scores on this scale might have been artificially elevated skewing the observed association (de Medeiros et al., 2020). Furthermore, institutionalized older adults frequently experience isolation and disability (Li W. et al., 2026; Melzer et al., 1999) whose objective quality of life might be lower (Silva e Farias et al., 2020). Yet this objective quality of life might not be fully captured due to the items focused on basic needs in this scale, as these items might skew the results while not realistically reflecting the psychological and social dimensions of quality of life that might be affected due to institutionalization. These results suggest that the observed association might have been skewed and may not represent a genuine relationship between self-directed ageism and quality of life. However, after adjusting for covariates in the regression model, higher levels of quality of life predicted lower levels of self-directed ageism, as discussed further below.

As for attitudes toward older adults, negative attitudes towards older people were positively associated with self-directed ageism. This finding was expected and paralleled previous findings that also indicated that negative ageist attitudes are positively linked with self-directed ageism (Scrivano and Jarrott, 2023). It also emerged as a significant predictor in the regression model, holding up even after controlling for all the other variables. Taken together, these findings provide majority support for convergent validity, while the inconsistent findings regarding quality of life suggest the need for cautious interpretation.

Regarding sociodemographic and health-related characteristics, several were significantly associated with self-directed ageism in the present sample. Higher levels of self-directed ageism were observed among participants aged 70–79 and more than 80 years compared to the younger age groups. The 70–79 age group reported the highest levels of self-directed ageism in the present study, parallel to Henry et al. (2023), the present results also seem to point to self-directed ageism to be intensifying with age. In terms of gender differences, no significant difference was observed in the bivariate analyses. Females emerged as a significant predictor in the regression model presenting higher levels of self-directed ageism for females after controlling for the other variables. This finding aligns with a phenomenon defined as double jeopardy where older women encounter both gender and age-based discriminations possibly amplifying the intensity of internalized ageist beliefs (Saadati, 2024; Shakeri and North, 2025).

Living situation was the strongest predictor of self-directed ageism, with nursing home residents being the strongest compared to people living with family members or by themselves. This is in line with Xue et al. (2025) where it was found that such nursing home environments might reduce older adults’ sense of autonomy which fosters higher levels of self-directed ageism. This seems to be concerning especially for the Lebanese context since Younes and Rajha (2025) has already documented ageist attitudes amongst healthcare workers further explaining this finding since nursing home environments might reinforce self-directed ageism.

High levels of self-directed ageism were also linked to the presence of health problems and financial dissatisfaction with financial situation being a significant predictor for self-directed ageism which is in line with previous studies (Ayalon and Cohn-Schwartz, 2021; Christian and Halliday, 2025). The pattern for financial satisfaction wasn’t linear as the ‘not satisfied’ group reported the highest levels rather than the ones reporting the lowest satisfaction (only *n* = 15 in “not at all satisfied”). The link between self-directed ageism and health problems is parallel with previous literature (Henry et al., 2024; Tully-Wilson et al., 2021). While education and employment status were significantly associated with self-directed ageism in the bivariate analysis, neither of these two emerged as a significant predictor in the regression model. Finally, after controlling for covariates, higher levels of quality of life were associated with lower levels of self-directed ageism, suggesting that the initial positive bivariate association might’ve been influenced by other psychosocial factors included in the model which is in line with (Vanleerberghe et al., 2024).

### Strengths, limitations, and future directions

The present study’s main strengths are that it presents the first validated Arabic tool that allows the capturing of self-directed ageism, addressing a significant gap in the literature amongst Arabic-speaking populations. The use of both exploratory and confirmatory analysis provides a thorough examination of the scale’s structural validity, while also assessing the convergent validity of the scale by using multiple theoretically relevant constructs to ensure it.

However, several limitations of the present study have to be acknowledged, the first one being that the sample had a significant proportion of nursing home residents and the minority being community-dwelling older adults, which does not realistically reflect the actual Lebanese demographic, limiting generalizability to the whole Lebanese demographic. Additionally, the high proportion of institutionalized participants may have influenced the psychometric properties obtained, as this subgroup may differ from community-dwelling older adults in health status, cognitive functioning, and level of independence, with institutionalization and reduced autonomy due to age potentially shaping how they perceive the aging process, and in turn how self-directed ageism is expressed on the scale.

Secondly, convenience sampling might have introduced selection bias since participants were not randomly selected, which further limits the representativeness of these present findings.

Another limitation concerns the use of different data collection modes, where institutionalized participants were administered the questionnaire in person at nursing homes while community-dwelling participants completed it online via Google Forms, which may have introduced mode-related bias and should be considered when interpreting differences by living situation.

Lastly, as with all studies relying on self-report measures, response bias should be considered, which might influence the accuracy of responses.

Future research should aim to extend the validation of the Arabic self-directed ageism scale across different populations that might be particularly vulnerable to self-directed ageism, such as older adults living with frailty (Vanleerberghe et al., 2024) or refugees facing multiple layers of discrimination which might shape self-directed ageism differently (Abi Chahine and Kienzler, 2022) while also applying this scale in interventions to measure the outcomes helping with identifying which types of interventions are most effective in reducing internalized ageist beliefs. Additionally, future validation studies should examine factorial invariance across sex, age groups, and residential settings to confirm that the scale measures self-directed ageism consistently across these subgroups. Longitudinal studies assessing the temporal stability of the instrument and its sensitivity to change would also strengthen the evidence supporting its use, particularly in intervention contexts where detecting meaningful change over time is essential.

## Conclusion

To conclude, the present results provide preliminary evidence for the reliability and validity of the Arabic version of the self-directed ageism scale among older Lebanese adults. The scale demonstrated satisfactory structural and convergent validity in this sample, supporting its potential utility for quantitatively assessing self-directed ageism. This scale was translated into the classic Arabic language, allowing it to be used in other Arabic-speaking countries. The present validation fills an important research gap, paving the way for rigorous quantitative research on self-directed Ageism in Arab societies. Beyond its psychometric contribution, the availability of a validated Arabic tool holds practical value, as it may support the development and evaluation of intervention programs targeting self-directed ageism, inform clinical practice by enabling screening for internalized ageist beliefs, and facilitate public health initiatives and epidemiological research on aging across the MENA region, ultimately helping to promote healthier and more positive aging experiences.

## Data Availability

The data presented in this study are available upon request from the corresponding author due to institutional ethical and legal reasons.
