# Supplementary material for: Psychometric validation of the Arabic self-directed ageism scale among Lebanese middle-aged and older adults
Source: Front Psychol. 2026 Sep 16;17:1911928. doi: 10.3389/fpsyg.2026.1911928 (PMC13624070; doi:10.3389/fpsyg.2026.1911928)
Supplement: Supplementary file 1 [file Table_1.docx]

| **Supplementary Table S1. Pearson Correlation Matrix Between Self-Ageism and Scale Items** | | | | | | | | | | | |
| --- | --- | --- | --- | --- | --- | --- | --- | --- | --- | --- | --- |
| **Variable** | **Self-directed ageism total scale** | **item 1** | **item 2** | **item 3** | **item 4** | **item 5** | **item 6** | **item 7** | **item 8** | **item 9** | **item 10** |
| **item 1** | .789** | - |  |  |  |  |  |  |  |  |  |
| **item 2** | .702** | .629** | - |  |  |  |  |  |  |  |  |
| **item 3** | .844** | .691** | .617** | - |  |  |  |  |  |  |  |
| **item 4** | .860** | .635** | .508** | .691** | - |  |  |  |  |  |  |
| **item 5** | .909** | .686** | .593** | .735** | .825** | - |  |  |  |  |  |
| **item 6** | .863** | .655** | .558** | .664** | .710** | .818** | - |  |  |  |  |
| **item 7** | .791** | .566** | .473** | .635** | .686** | .702** | .673** | - |  |  |  |
| **item 8** | .615** | .427** | .413** | .492** | .449** | .509** | .587** | .400** | - |  |  |
| **item 9** | .890** | .623** | .560** | .741** | .805** | .818** | .757** | .692** | .526** | - |  |
| **item 10** | .783** | .530** | .458** | .627** | .672** | .705** | .632** | .580** | .365** | .695** | - |
| **item 11** | .580** | .428** | .269** | .401** | .415** | .440** | .466** | .397** | .243** | .461** | .407** |
| **Correlation is significant at the 0.01 level (2-tailed). | | | | | | | | | | | |
