# Supplementary material for: Psychometric validation of the Arabic self-directed ageism scale among Lebanese middle-aged and older adults
Source: Front Psychol. 2026 Sep 16;17:1911928. doi: 10.3389/fpsyg.2026.1911928 (PMC13624070; doi:10.3389/fpsyg.2026.1911928)
Supplement: Supplementary file 2 [file Table_2.docx]

Supplementary file

| **Table S2. Bivariate and Partial Correlations Between Self-Directed Ageism and Quality of Life** | |
| --- | --- |
| **Analysis** | **Association between self-directed ageism and QOL** |
| **Bivariate Spearman correlation** | **r = 0.288, p < 0.001** |
| **Partial correlation**, adjusted for the covariates | **r = −0.098, p = 0.155** |
| Covariates: age, gender, education, financial situation, health problems, self-esteem, attitudes toward older people, employment status, and current housing situation. | |
